# Accuracy of guided surgery using the silicon impression and digital impression method for the mandibular free end: a comparative study

**DOI:** 10.1186/s40729-020-00281-z

**Published:** 2021-01-12

**Authors:** Koudai Nagata, Kei Fuchigami, Noriyuki Hoshi, Mihoko Atsumi, Katsuhiko Kimoto, Hiromasa Kawana

**Affiliations:** 1 Department of Oral and Maxillofacial Implantology, Kanagawa Dental University, 82 Inaoka-cho, Yokosuka, 238-8580 Japan; 2 Division of Prosthodontics and Oral Implantology, Department of Oral Interdisciplinary Medicine, Graduate School of Dentistry, Kanagawa Dental University, Yokosuka, Japan

**Keywords:** Computer-aided surgery, Dental implant, Digital workflow, Guided surgery, Implant impression, Intraoral scanner

## Abstract

**Background:**

Implant treatment using guided surgery is becoming widespread in clinical dental practice. Furthermore, the development of digital technology has enabled the use of intraoral scanners (IOSs) to fabricate surgical guide plates. The objective of this study was to compare the accuracy of guided surgery using the silicone impression method with a three-dimensional (3D) scanner and the digital impression method with IOS for one side of the mandibular free end. In addition, we compared the accuracy of tooth-supported vs tooth/mucosa-supported surgical guide plates.

**Results:**

The accuracy of the tooth-supported surgical guide plate using the new IOS method instead of the method of obtaining impressions with conventional silicone resulted in better measurements of 3D deviation at the crest, 3D deviation at the apex, and angular deviation. In terms of the accuracy of the tooth/mucosa-supported surgical guide plate, there were no significant differences in all measurements. The surgical guide plate using an IOS and the tooth/mucosa-supported surgical guide plate may enable more accurate guided surgery.

**Conclusion:**

Tooth/mucosa-supported guided surgery involving preparation with an IOS may result in more accurate implant surgery.

## Background

Implant treatment using guided surgery has recently become widespread; it is now possible to apply this technique to a wide range of conditions, from a single missing tooth to edentulous jaws. Furthermore, the development of digital technology has enabled the fabrication of a surgical guide plate by converting intraoral images obtained with an intraoral scanner (IOS) to standard triangulated language (STL) data and by subsequently superimposing these data with those of cone beam computed tomography (CBCT) and Digital Imaging and Communications in Medicine (DICOM) [1–3]. However, it has not been fully verified whether these new digital technologies have superior accuracy compared to the conventional techniques. Safety concerns make it difficult to adopt a new technology with poor accuracy. Moreover, the accuracy of surgical guide plates is particularly important due to their direct relation to the surgery. Thus, we compared the accuracy of the conventional method, where a silicone impression was obtained using a 3D scanner by reading the plaster model of the right mandibular free end, to that of a new method, where a digital impression was obtained using an IOS. In both techniques, the impression obtained was converted to STL data and subsequently superimposed with DICOM to create a surgical guide plate. Although there are many previous reports on guided surgery, only few have compared the accuracy of tooth-supported and tooth/mucosa-supported surgical guides; therefore, we also compared the accuracy of these two techniques.

## Methods

The following experimental plans were formulated to compare the accuracy of the conventional method to that of the new method. Other than the impression acquisition technique, both methods followed the same protocol for the conversion to STL data, CBCT imaging, DICOM data acquisition, superimposition on simulation software, surgical guide plate fabrication, and guided implant placement on the plaster model. Additionally, we examined whether using a tooth-supported device or a tooth/mucosa-supported device would result in different outcomes. As our study was performed using models, no institutional review board approval was required.

### Targets

The target of the study was a jaw model with a right mandibular free-end defect. The model had three consecutive missing teeth from the right second premolar to the second molar (45–47, FDI two-digital notation). It was presumed that the implant would be placed at two locations: at the right second premolar and at the second molar. The existing jaw model for mucosal implant training with contrast media (NISSIN, Tokyo, Japan) was used as the jaw model.

### Fabrication of the surgical guide plate

First, the target jaw model was imaged using CBCT (3DX®; Morita, Tokyo, Japan), and the obtained DICOM data were read using simulation software (coDiagnostiX®; Dental Wings, Montreal). The STL data were acquired for both the conventional method and the new method; based on the use of different IOSs, the new method was further divided into two groups, explained in the subsequent text.

### Si group (conventional method)

For the silicone impression material group (Si group), a plaster model was fabricated from a silicone impression (Aquasil Ultra®; Dentsply Sirona, York, USA), which was then scanned with a 3D scanner (Ceramill Map 400®; Amann Girrbach, Wien, Austria). The plaster model was prepared with the standard recommended technique of using a hard-type die stone (New Fujirock®; GC, Tokyo, Japan), with a water mixing ratio of 0.20 (powder, 100 g; water, 20 mL). The poured plaster model was then left to set and harden in a closed box for 11 min.

### Tri group (new method)

For the Trios group (Tri group), a digital impression was obtained using the IOS Trios 3® (3 shape; Copenhagen, Denmark) and then scanned.

### CS group (new method)

For the CS3600® (Carestream Dental, Atlanta, USA) group (CS group), a digital impression was obtained using the IOS CS3600® (Carestream Dental) and then scanned.

Digital wax-ups were performed on STL data obtained from the three groups of STLs with reference to opposing models using a computer-aided design (Exocad®; Exocad, Berlin, Germany), which is capable of dental prosthetic design.

**Fig. 1:**
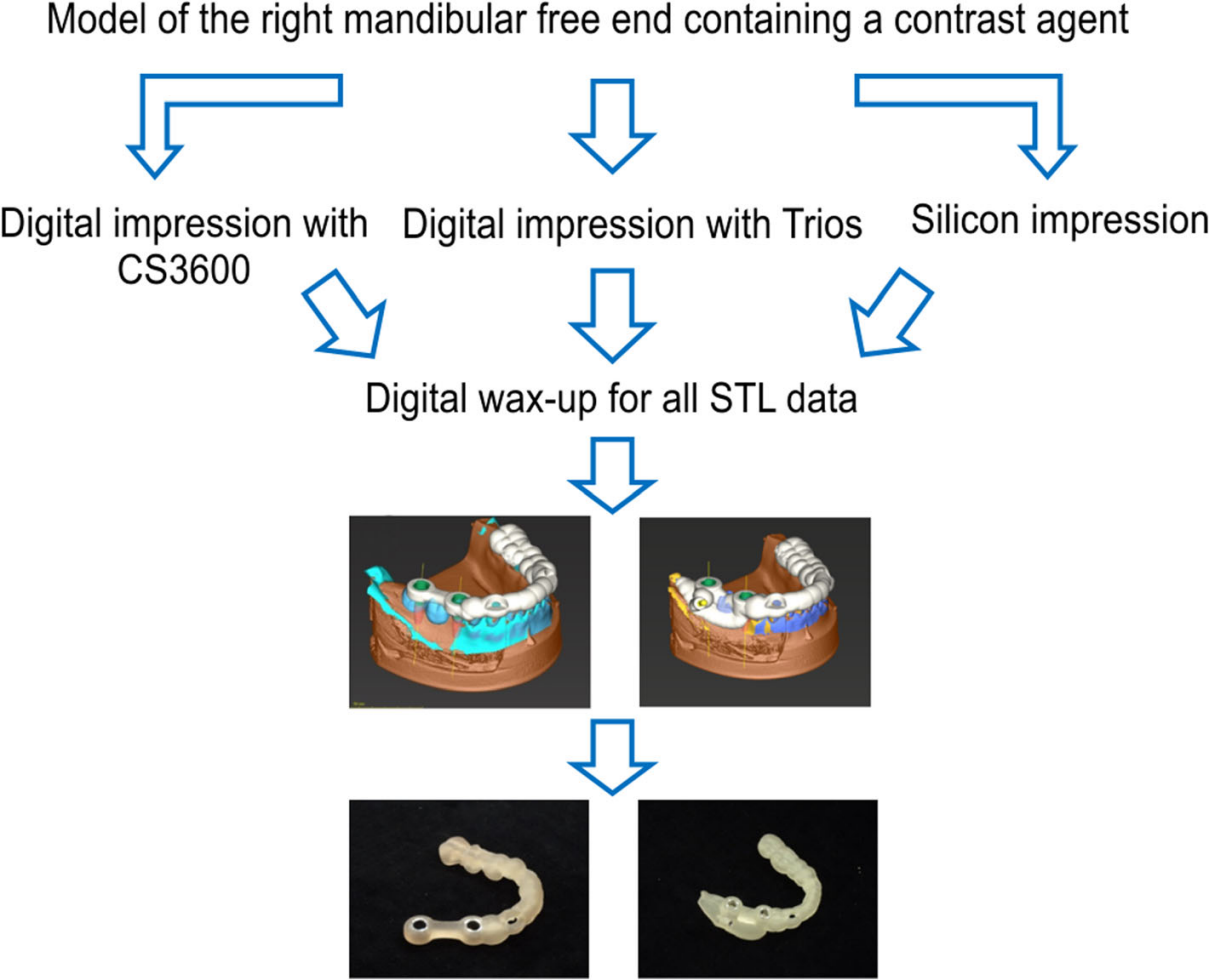
Flow chart of the fabrication of surgical guide plates. Tooth-supported surgical guide plate and tooth/mucosa-supported surgical guide plate

A tooth-supported surgical guide plate and a tooth/mucosa-supported surgical guide plate were then created from the simulated data (Fig. 1).

### Modeling for implantation

**Fig. 2:**
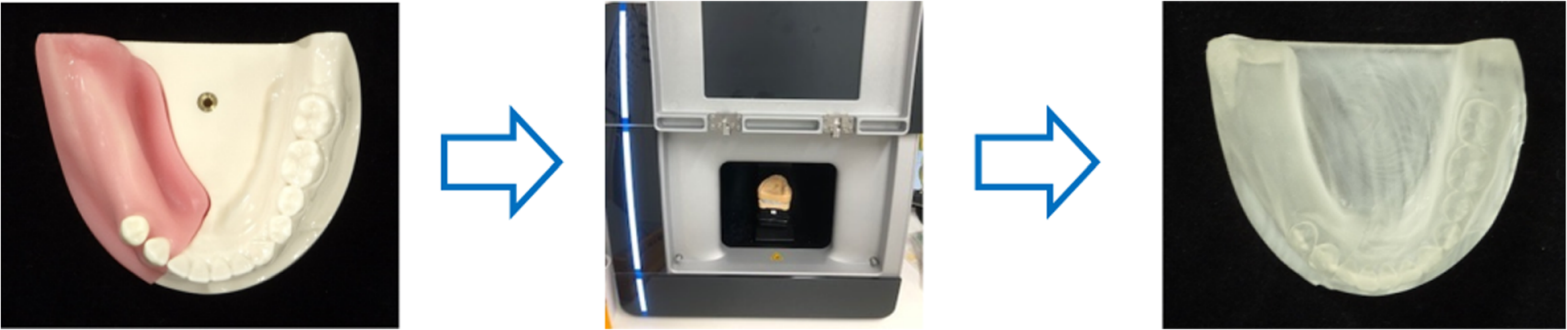
Flow chart of resin model production. Eighteen resin models were created from three-dimensional (3D) scans

Eighteen resin models were fabricated from the mandibular unilateral free end models using a 3D printer (Form2®, Formlabs, Washington, DC, USA) and a 3D scanner (Ceramill Map400®, Amann Girrbach, Wien, Austria) (Fig. 2).

### Implant implantation method

**Fig. 3:**
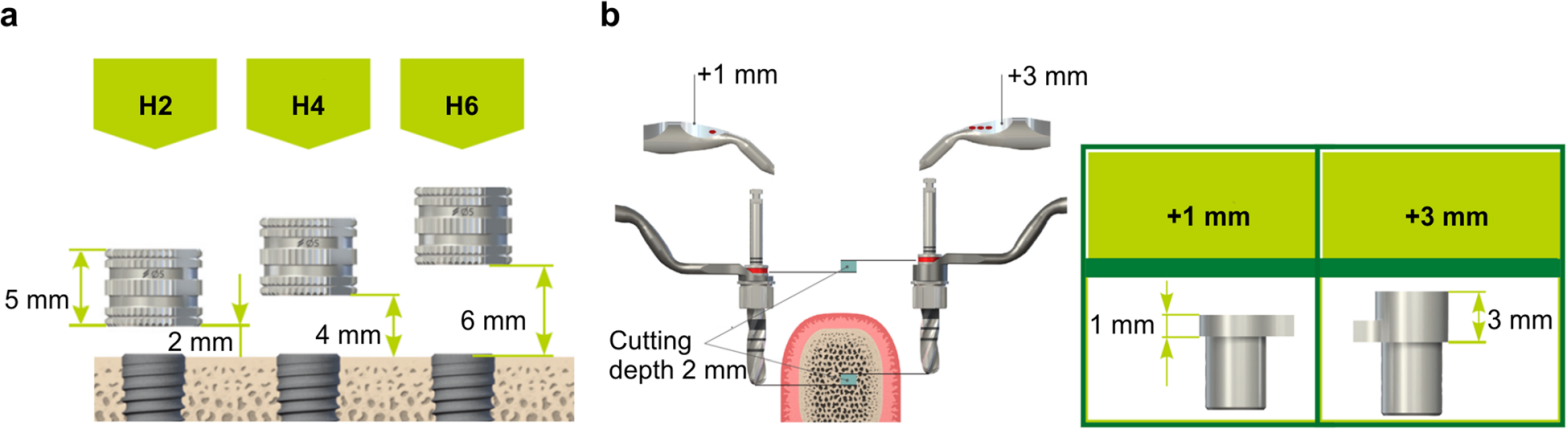
Guide system. **a** Sleeve height (*H*). **b** Key height. Modified and reprinted with permission from Straumann Japan (Tokyo)

The implants were embedded in the finished resin models for all the three groups; each group was further subdivided based on the use of tooth-supported or tooth/mucosa-supported surgical guide plates. The embedding sites were set at 45 and 47, using the digital wax-up as the reference. A fully guided implant surgery was performed following drill steps determined by the simulation software (key height, 1 mm; drill, long; sleeve height, 4 mm; offset, 0.08 mm). The offset value was set following the manufacturer’s instructions. The implants used for the study were Straumann® BLT/RCφ4.1 × 10 (Straumann, Basel, Switzerland). Each surgical guide plate was used for three resin models. Tooth/mucosa-supported surgical guide plates were fixed behind 47 with a single anchor pin. All implant placements were performed by the same physician (Fig. 3). An assistant held the surgical guide plate during implantation.

### Measurement methods

**Fig. 4:**
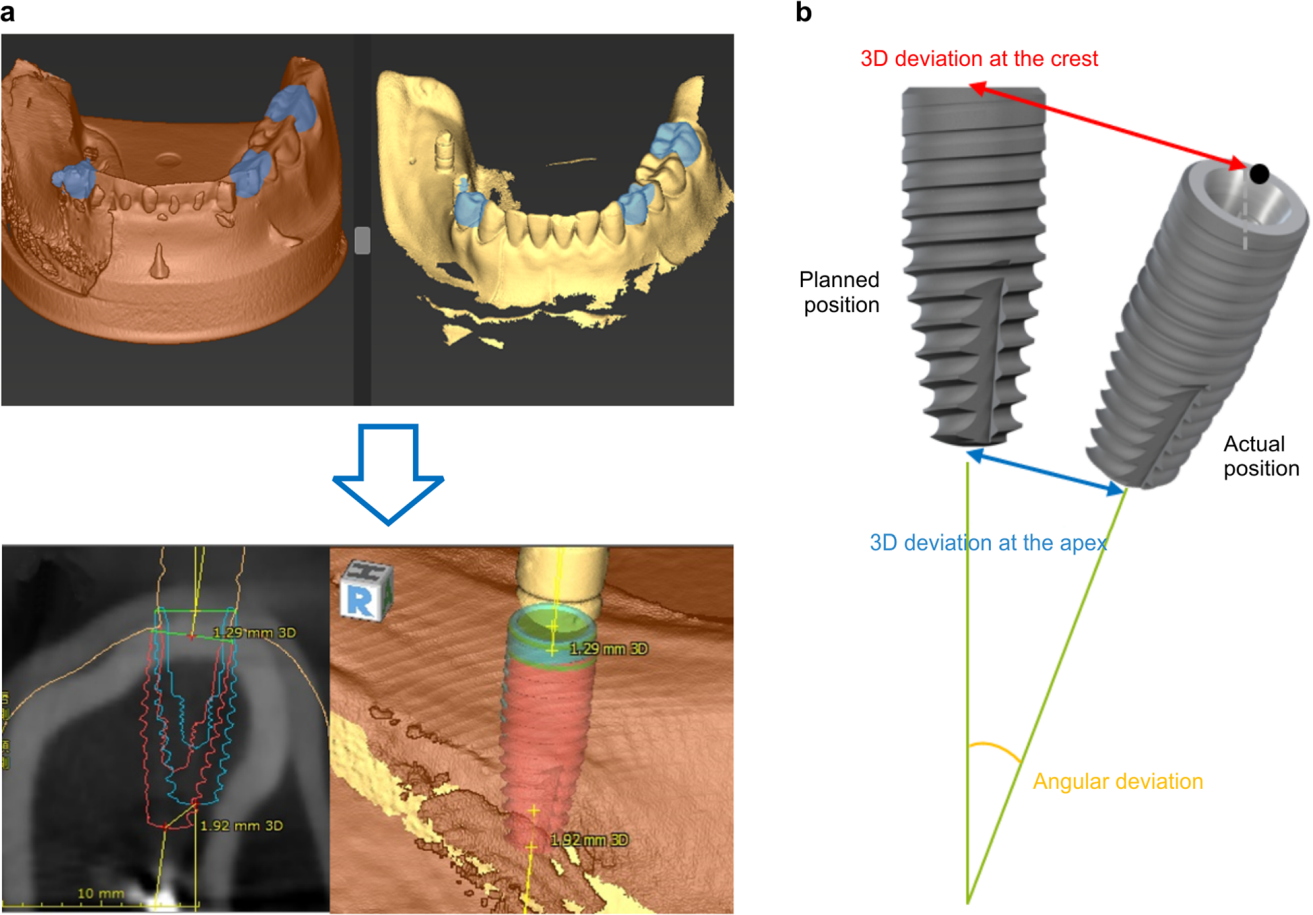
Measurement of errors. **a** After implantation, STL data of the resin model equipped with a scan body are superimposed on coDiagnostiX simulation data. Accuracy was measured using the treatment evaluation tool. Blue: implantation position set using simulation. Red: implanted position. **b** Evaluation of deviation measurements

A scan body for Straumann RC (CARES Scan CS2®; Straumann) was mounted on the implant embedded in the resin model and read by a 3D scanner. The data were converted to STL. Then, using the built-in treatment evaluation tool of the simulation software (coDiagnostiX), the accuracy was calculated by superimposing the planned implantation position set at the time of surgical guide plate fabrication with the actual implantation position. All accuracies were calculated automatically. The measurements were recorded at three locations: 3D deviation at the crest, 3D deviation at the apex, and angular deviation. All three measurements were taken for each model, and the average value was used as the result (Fig. 4).

### Statistical analysis

The Tukey-Kramer method was used to compare both the accuracy of the surgical guides made by each impression method and the accuracy of the tooth-supported and tooth/mucosa-supported surgical guides. Statistical analyses were done using BellCurve for Excel (Social Survey Research Information Co., Ltd., Tokyo, Japan). The differences with a *P* value < 0.05 were considered statistically significant.

## Results

### Accuracy of the tooth-supported surgical guide (Fig. 5)

We compared the three-dimensional (3D) deviations at the crest in 45 between the Si, CS, and Tri groups. The results indicated significant differences between the Si and CS groups and between the Si and Tri groups (*P* < 0.001). Further, 3D deviations at the crest in 47 for the Si, CS, and Tri groups were 1.4 ± 0.41 mm, 0.97 ± 0.15 mm, and 1.06 ± 0.48 mm, respectively. The results indicated no significant differences between the groups. Next, the 3D deviations at the apex in 45 for the Si, CS, and Tri groups were 1.84 ± 0.18 mm, 1.07 ± 0.32 mm, and 1.11 ± 0.2 mm, respectively. The results indicated significant differences between the Si and CS groups and between the Si and Tri groups. The 3D deviations at the apex in 47 for the Si, CS, and Tri groups were 1.89 ± 0.22 mm, 1.1 ± 0.17 mm, and 1.38 ± 0.25 mm, respectively. The results indicated significant differences between the Si and CS groups and between the Si and Tri groups (*P* < 0.001).

**Fig. 5:**
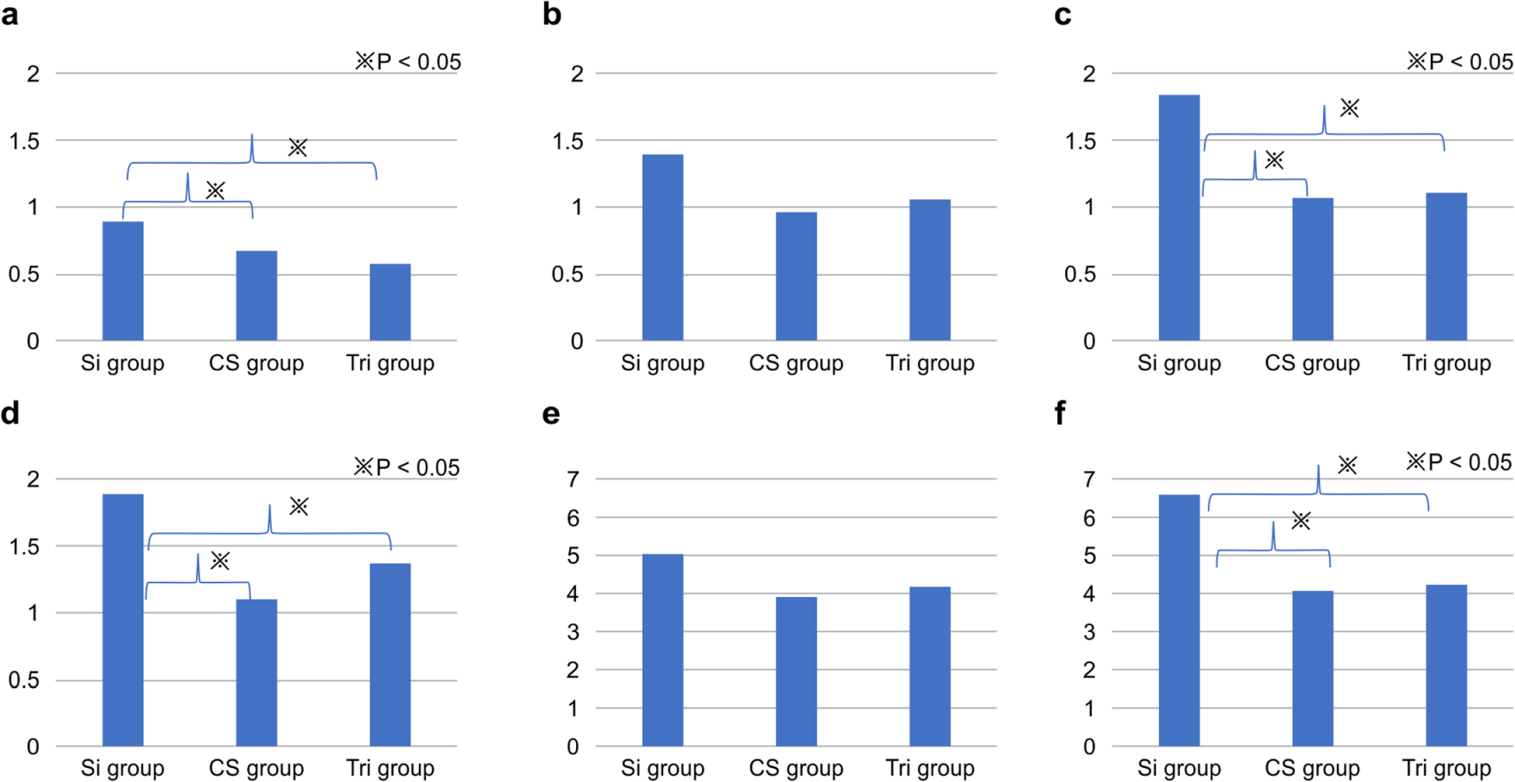
Accuracy of tooth-supported surgical guides. **a** Three-dimensional (3D) deviation at the crest in 45. **b** 3D deviation at the crest in 47. **c** 3D deviation at the apex in 45. **d** 3D deviation at the apex in 47. **e** Angular deviation in 45. **f** Angular deviation in 47

Angular deviations in 45 for the Si, CS, and Tri groups were 5.08 ± 1.33°, 3.91 ± 0.52°, and 4.19 ± 1.9°, respectively. The results indicated no significant differences. Angular deviations in 47 for the Si, CS, and Tri groups were 6.56 ± 0.44°, 4.06 ± 1.06°, and 4.21 ± 0.44°, respectively. The results indicated significant differences between the Si and CS groups and between the Si and Tri groups (*P* < 0.001).

### Accuracy of the tooth/mucosa-supported surgical guide (Fig. 6)

The 3D deviations at the crest in 45 for the Si, CS, and Tri groups were 0.44 ± 0.25 mm, 0.48 ± 0.1 mm, and 0.42 ± 0.13 mm, respectively. The results indicated no significant differences. The 3D deviations at the crest in 47 for the Si, CS, and Tri groups were 0.84 ± 0.35 mm, 0.53 ± 0.11 mm, and 0.6 ± 0.13 mm, respectively. The results indicated no significant differences. The 3D deviations at the apex in 45 for the Si, CS, and Tri groups were 0.88 ± 0.06 mm, 0.84 ± 0.22 mm, and 0.79 ± 0.14 mm, respectively. The results indicated no significant differences. The 3D deviations at the apex in 47 for the Si, CS, and Tri groups were 0.85 ± 0.39 mm, 0.65 ± 0.07 mm, and 0.66 ± 0.05 mm, respectively. The results indicated no significant differences.

**Fig. 6:**
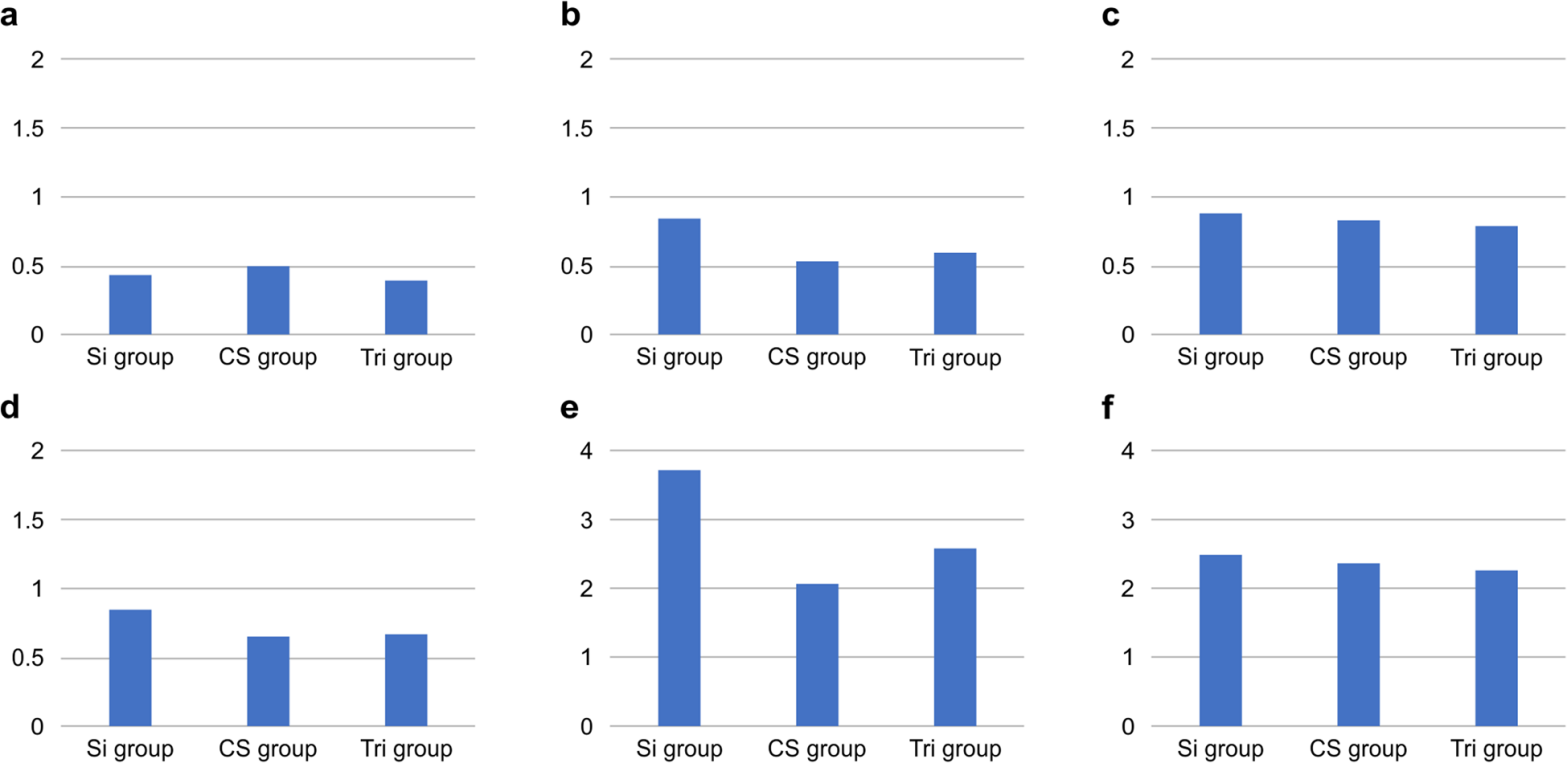
Accuracy of tooth/mucosa-supported surgical guides. **a** Three-dimensional (3D) deviation at the crest in 45. **b** 3D deviation at the crest in 47. **c** 3D deviation at the apex in 45. **d** 3D deviation at the apex in 47. **e** Angular deviation in 45. **f** Angular deviation in 47

Angular deviations in 45 for the Si, CS, and Tri groups were 3.72 ± 1.23°, 2.11 ± 1.68°, and 2.59 ± 0.41°, respectively. The results indicated no significant differences. Angular deviations in 47 for the Si, CS, and Tri groups were 2.49 ± 1.48°, 2.36 ± 1.41°, and 2.26 ± 0.37°, respectively. The results indicated no significant differences.

### Comparison of the accuracy of tooth-supported guided surgery and tooth/mucosa-supported guided surgery (Fig. 7)

Regarding the 3D deviation at the crest in 45, significant differences were found between the Si and CS groups. For 3D deviation at the crest in 47, significant differences were found in all three groups. Furthermore, significant differences in 3D deviation at the apex in 45 were evident in the Si and Tri groups. Finally, for 3D deviation at the apex in 47, all groups demonstrated significant within-group differences (*P* < 0.001).

**Fig. 7:**
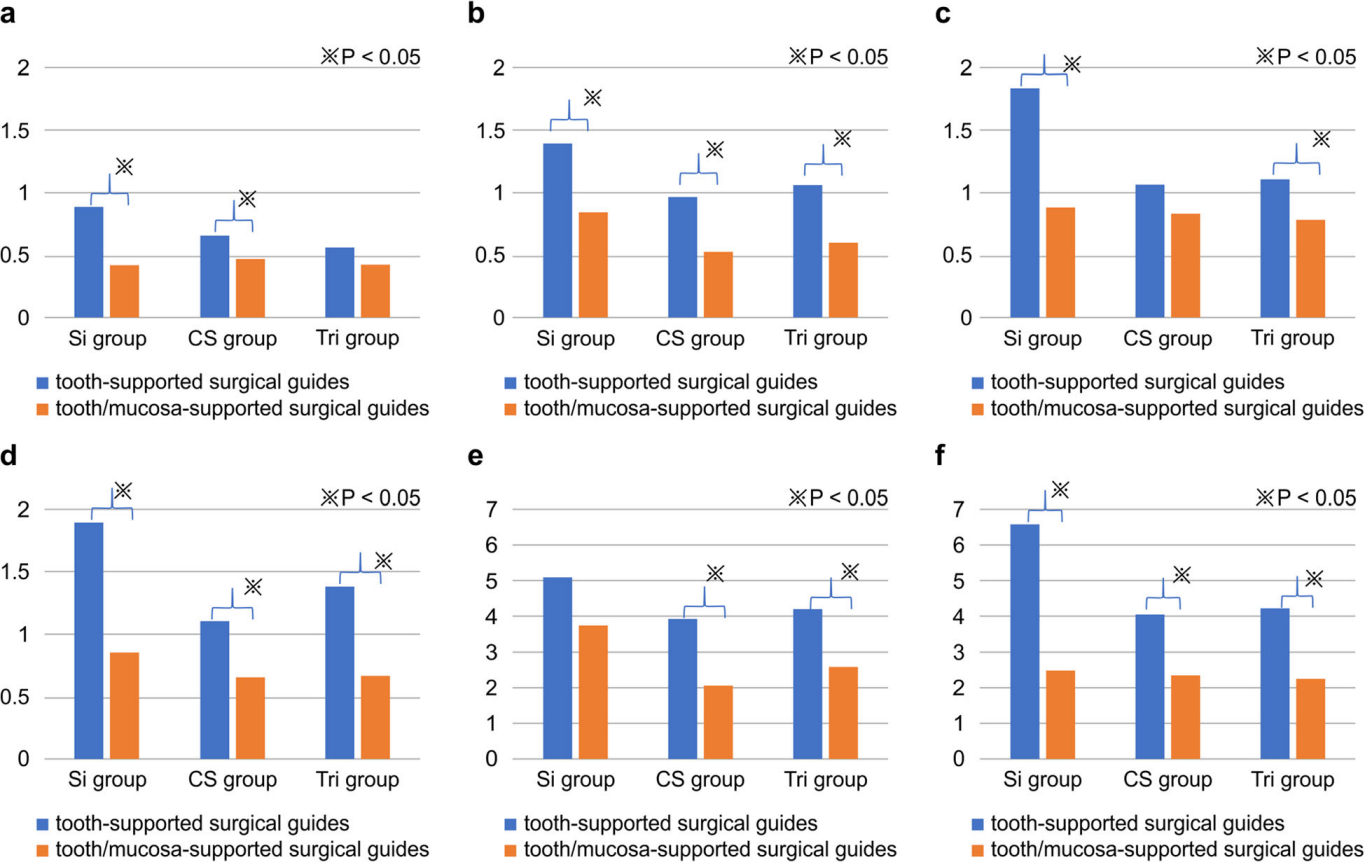
Comparison of tooth-supported and tooth/mucosa-supported guided surgeries. **a** Three-dimensional (3D) deviation at the crest in 45. **b** 3D deviation at the crest in 47. **c** 3D deviation at the apex in 45. **d** 3D deviation at the apex in 47. **e** Angular deviation in 45. **f** Angular deviation in 47

Regarding the angular deviation in 45, significant differences were found between the CS and Tri groups. Furthermore, the within-group assessment demonstrated significant differences in angular deviation in 47.

## Discussion

According to the evaluation of guided surgery using the tooth-supported surgical guide plate, the new method using an IOS had better measurement (3D deviation at the crest, 3D deviation at the apex, and angular deviation) accuracy than the conventional method using silicone. A comparison of the tooth-supported surgical guide plate and the tooth/mucosa-supported surgical guide plate indicated a good accuracy for all measurements taken using the tooth/mucosa-supported surgical guide plate.

To fabricate a surgical guide plate, it is necessary to make a precise impression with the silicone impression material, plaster the model, perform a wax-up, read the model with a 3D scanner, and convert it to STL data. However, there have been concerns regarding the deformation of the impression material, expansion of the plaster, and accuracy of the 3D scanner [4–7]. The steps involved in the conventional process could be omitted by using an IOS, thereby reducing these errors [8, 9]. The results of this study also suggested the superiority of the IOS over the conventional process.

Although there have been many reports on the accuracy of guided surgery, few have fabricated a surgical guide plate using an IOS and measured the accuracy. Derksen et al. used a coDiagnostiX treatment evaluation tool and measured the accuracy of guided surgery using the same method as ours. They prepared surgical guide plates using an IOS and measured the accuracy in 66 patients with partial edentulous jaws using 145 implants. They reported an error of 0.75 mm (± 0.34) at the starting point, 1.06 mm (± 0.44) at the tip, and 2.72° (± 1.42) for the angle. Moreover, superimposing DICOM data and STL data improved the accuracy in case of seven or more unrestored teeth [10]. Similar results were observed in our study while using the IOS.

Smitkarn et al. compared guided surgery using an IOS and free-hand surgery in 42 patients with a single missing tooth. The method employed was the same as in our study. They observed an error of 0.9 mm vs 1.3 mm at the starting point, 1.2 mm vs 2.2 mm at the tip, and 2.8° vs 7° in the accuracy of guided surgery and the free-hand group, respectively. Similar to the results of this study and Derksen et al.’s, they also found guided surgery to be superior [11].

Behneke et al. compared the accuracy of guided surgery in 52 patients with partial edentulous jaws using 132 implants; they reported a higher accuracy with single tooth loss than with partial loss [12]. A systematic review by Tahmaseb et al. examined the accuracy of guided surgery in 20 articles reporting on 471 patients and 2238 implants. They found an average error of 1.2 mm at the starting point, 1.4 mm at the tip, and 3.5° for the angle, thereby highlighting the importance of providing a safety margin of 2 mm. They reported better accuracy for a partial defect than for an edentulous jaw [13]. In our study, guided surgery using an IOS resulted in good accuracy for all measurement points (starting point, tip, and angle).

Tahmaseb et al., who studied implantation using guided surgery, reported an error of 1.34 mm at the starting point and 5.13° for the angle in the flap group that was treated with an incision and detachment of the mucosa at the implantation site; the flapless group had an error of 1.01 mm at the starting point and 3.42° for the angle, indicating better accuracy with the flapless method [14]. El Kholy et al. reported a greater angle error with a longer drilling distance under the guide sleeve [15]. A systematic review by Bover-Ramos et al. compared the accuracy of the full guide and half guide for 3011 implants in 34 articles; they found an error of 1.23 ± 0.1 mm vs 1.91 ± 0.23 mm at the implant tip, a vertical error of 0.62 ± 0.08 mm vs 0.83 ± 0.23 mm, and an angle error of 3.13 ± 0.23° vs 4.30 ± 0.73° with the full guide and half guide, respectively [16]. In the flapless guided surgery, the surgical guide is placed on the mucous membrane and the sleeve is positioned separately from the bony surface. Therefore, for a more precise guided surgery, the guide sleeve should be shortened, and a full guide should be used, which would reduce errors in the implantation position of the implant body. However, with the surgical guide fabrication method used in this study, sleeve heights of 2, 4, or 6 mm can be used. Setting the guide sleeve to 2 mm could enable more accurate guided surgery.

Among the reports on the accuracy of guided surgery, few have compared the tooth-supported surgical guide and the tooth/mucosa-supported surgical guide for free-end defects. El Kholy et al. evaluated the accuracy of guided surgery with the surgical guide supported by a full arch and four, three, and two teeth adjacent to the intermediary single tooth defects. They reported good accuracy, without any error, for the full arch and four adjacent teeth and noted larger errors in the tooth-supported surgical guide for free-end defects than for intermediary single tooth defects [17].

Toyoshima et al. in their study on the accuracy of guided surgery using a tooth-supported surgical guide prepared with the silicon impression method for the right mandibular free-end defect model noted a larger error in the second molar than in the second premolar [18]. We also observed similar results, with larger errors noted in guided surgery using a tooth-supported surgical guide for free-end defects. One of the drawbacks of surgical guides made from silicon impressions, especially in the case of molars, is the problem arising due to the amount of required mouth opening and guide deflection [13, 19]; however, this problem can be mitigated with the use of IOS and a tooth/mucosa-supported surgical guide plate. Here, we found that guided surgery using a tooth/mucosa-supported surgical guide prepared with an IOS reflected the preoperative setting position with greater accuracy. The digital impression by the IOS and posterior support by the anchor pins may have stabilized the surgical guide plate and led to a good result. However, when a dental mucosa-supported surgical guide plate was used, the mucosa after the flap may have interfered with the fit of the surgical guide plate. It is therefore necessary to eliminate the flap and check the fit of the anchor pin; alternatively, flapless surgery can be pursued.

There are many reports on the accuracy of guided surgery, but most have examined conventional methods using silicone impression materials. Therefore, more studies are warranted to determine the accuracy of guided surgery using the IOS.

A limitation of our study is that it is only based on a model. Hence, additional clinical trials are needed.

## Conclusion

This study found that surgical guides for mandibular free-end implant surgery had smaller errors when fabricated with the digital impression method using the IOS than when produced with the conventional method using silicon. Guided surgery resulted in a smaller error with the use of tooth/mucosa-supported surgical guides than with tooth-supported surgical guides. These results suggest that guided surgery using tooth/mucosa-supported surgical guides prepared with an IOS contributes to more accurate implant surgery. We assumed three consecutive tooth defects at the free end of the mandible but would like to examine the application of multiple tooth defects in future studies.

## Data Availability

The datasets used and/or analyzed during the current study are available from the corresponding author on reasonable request.

## Abbreviations

3D: Three-dimensional
CBCT: Cone beam computed tomography
DICOM: Digital Imaging and Communications in Medicine
IOS: Intraoral scanner
STL: Standard triangulated language
